# With Our Editors

**Published:** 1906-07-15

**Authors:** 


### WITH OUR EDITORS.

**A REMEDY FOR THE TROUBLES IN THE NATIONAL DENTAL ASSOCIATION.**

The Editor of *Items of Interest* was recently criticised by a gentleman for whom he has high esteem, as "a man who finds fault but offers no remedy." The Editor might call to mind one or two remedies proposed in these pages which have proven efficacious, but that would be retrospective. The question of the hour is the dissatisfaction in the National Dental Association as at present constituted, and the remedy therefore is the remedy sought.

THE PRESENT DIFFICULTY ANALIZED.—Before any disease can be scientifically treated it must be fully comprehended. Let us attempt a diagnosis of the ailment which threatens to disrupt the National; the word disrupt is used advisedly. It is easy enough to scream "politics" but if you ask a dentist, "Who are the politicians?" he replies: "Why, you are one of them." Then both men deny the allegation. Since the word proves offensive, though really it is a quite proper word for proper men, let us omit it from the present discussion. It should be the loyal duty of every member of the National to strive to heal the breach if possible..

Let us then say that there are two prominent classes of men engaged in what we term "society work." These be the executive men; and the writers and clinicians, or scientific men. This classification is not an arbitrary one because many men belong to both classes; a few are even conspicuous workers in both.

Nevertheless we may, for the purpose of this argument, distinguish between the executive men, those that organize the program and the scientists, those that contribute the program. No society can be made successful or useful, without the existence and co-operation of both. These facts are so elemental that they will not be disputed. Let us now consider their practical application.

LOCAL SOCIETY WORK.—A local society is a body of dentists in a large city, or in an area including several smaller cities, associated together for mutual benefit. But as such men are neighbors, an essayist or clinician from their own membership is less able to instruct, than one from a distance. This is the constant aim of capable executives of local societies to obtain program material from distant points. Consequently the scientific element in the local society places that body so little under obligation, that it has scarcely any claim upon the offices, which naturally are given to the executives, towards whom the members do feel indebted. This is both praisworthy and proper. Nevertheless, the situation should never be abused. In a body of professional men, no man, however much he may have placed the society in his debt, should ever become an office seeker. When he does, he makes of himself a politician in the offensive sense.

State Society Work.—There is a little difference when we come to consider State Societies, though at best the principle is much the same, because a State Society is only a local society in a larger locality. True, because of this increased area, there is an increased chance to fill the program from the membership list. Practically, however, all executives of any experience know that the success of the annual session will largely depend upon the proportion of men from other states that may appear upon their announcements. Thus again the executive element has the larger claim upon the offices and usually occupy them.

In the selection of presidents for State Societies two modes of procedure have been successfully carried into effect. Where the state is definitely divided into districts, it is sometimes the rule to choose presidents from the various sections in rotation, but even then the man chosen is commonly one who has served in a lesser position. The presidency is his final reward for his executive work. In an undistricted state, in a certain well managed State Society, the new member is offered a place on some minor committee as soon as he is elected. If he accepts and becomes a worker, he is promoted year by year in accordance with his ability and willingness. Finally he reaches the Clinic Committee and then is "in line" for the presidency. After the Clinic Committee the the Vice-Presidency and then the Presidency. There has not been a contest over offices in that Society in twenty years. It is a smooth running effective machine, but there are no "politicians," because every man has an exactly even chance.

But when we reach the National Dental Association, the principle changes, and it is this fact that has not been recognized; it is because the National is and always has been managed just as a local or a state organization should be, that all the hue and cry against politicians has arisen. There are miner wrongs in the National's constitution, but these need not be considered just now. The major wrong is that the presidency is practically within the gift of the executive element, and that it has usually been alotted to one of themselves. This work does not excuse the executive men of wrong doing, because their work and their rewards in local and State bodies have them into the error of adopting as the motto on their escutcheon, "To the victor belongs the Spoils." Possibly this will be, so long as they will it to be. But so long as they do the National will never be what in might be; what it ought to be.

Have not the executive men benefited enough by the work of the scientific men in local and State societies, to stand ready to offer those that helped them to position and place, some meed of recognition in the National?

In the National, unlike the local or State society, there is no region beyond, from which to recoup the program. Essays and clinics must come from the scientific element within the society. Shall this element, those students and writers who have traveled to help the local societies, traveled to aid state societies, finally travel to and from the National without recognition?

THE REMEDY.—Often things are best seen in their truest light when viewed from a distance. Let us suppose a man in Europe, hearing for the first time of the National Dental Association of the United States. He notes the name of its president, and recalling that America is said to be the creator of the profession of dentistry, would it surprise you if he thought that the president of the National Dental Association must be the greatest dentist in America? Would it be true?

Why should it not be true? If the presidency is the apple of discord, which makes politicians of our executives, why not remove the presidency beyond the grasp of a mere executive man? We have admitted that a man may be both executive and scientific, thus we suggest no barrier against any member. But why not make the presidency of the National Dental Association the final reward and honor for high scientific attainment? If perchance such a student should prove a poor presiding officer, he would have three vice-presidents who might preside for him, and these could all come from the evecutive ranks. But it is the presidency, apparently, that makes men avaricious for office; then let us hang this apple on a higher tree.

How may this be done? Very simply. Let the president be elected by a two-thirds vote of the entire association, conducted by mail. Then let the incumbent be president year after year until some other man reaches such esteem among our members that two-thirds of us would name him for the office and the honor, without the suggestion of a folded piece of paper carrying a name, and secretly passed, as is commonly seen during the so-called informal ballot. Some will say, "Why, then, a man might be president for years." Well, why not, if no one equally prominent be found? Was not Judge Daly president of the National Geographical Society for over fifteen years?

Of course, the scheme is Utopian, but it would be effective. The mail ballot, even if a majority be deemed sufficient to elect, completely kills lobbying, and is it lobbying that causes us to whisper "politics."

If this remedy should be considered too radical, there be others less extreme which might be suggested, which indeed have been discussed. But why not try the radical cure at once?

In some cases of dysentery castor oil is the best medicine, yet many men prefer blackberry brandy. But the doctors know that the oil is better than the brandy.—June *Items of Interest.*

**THE ELDER THREE AMONG DENTAL COLLEGES.** As compared with seats of learning venerable through centuries of time, educational institutions with an historic back-ground of only fifty, sixty or sixty-five years can hardly be said to have survived the perils of childhood. Fifty years in the brief span of human life has been termed "the old age of youth, and the youth of old age;" and dentistry as a profession, is so young that a dental school which has survived to celebrate its fiftieth birth-day may, relatively speaking, fairly be said to have attained to the old age of its youth and entered upon the ever lessening youth of an ever increasing old age.

Among existing dental schools three have become thus relatively venerable: The Baltimore College of Dental Surgery, founded in 1840, the first dental college ever established and the oldest in the world; the Ohio College of Dental Surgery, founded in 1845, and the Pennsylvania College of Dental Surgery, founded in 1856, the latter, with fitting golden anniversary obserbvances, having just closed its fiftieth annual session.

This priority of entrance upon the institutional, as distinguished from the private training of dental students naturally gave to these schools some pre-eminence in dental college work. From one or the other of them several thousand graduates have received their dental education, and of these many have been active in establishing other of the numerous schools now in existence, or, from time to time have been called to positions in their respective faculties.

The founders of these older schools entered upon their educational work at a period when there was no apparent probability that it could to any adequate degree be made remunerative, but, on the contrary, with every possibility that, in addition to sacrifice of time, the undertaking might result in pecuniary loss. Hence the founding of the schools cannot be regarded as having been money-making schemes. For many years the classes were so small and, as compared with income, the necessary running expenses so great that demonstrators sometimes declined to accept advancement to professorships, because they could not afford to give up their fixed pay for the smaller income, or no income, of a faculty position. In view of these facts it is manifest that the pioneers in dental education can have been inspired only by a love for teaching and a desire for the advancement of their profession.

So impressionable is youth that students are not simply educated in the technical sense by their teacher, but often, unconscicusly to themselves, are profoundly influenced by their moral qualities. Thus the personality of the founders of a school becomes a transmitted influence, descending through successive generations of teachers and alumni, and in this way determining the dominant characteristics of the school itself.

From this, as well as from all other standpoints, it must be regarded as especially fortunate that all three of the elder schools were founded by men of sterling moral worth, as well as of high professional ability. Pre-eminently endowed with these qualities were Chapin A. Harris and Horace H. Hayden, through whose unselfish efforts for the advancement of dental education the first and oldest dental college in the world was established. In every way worthy of respect and esteem were Jesse W. Cook, Melancthon Rogers and James Taylor, active in the organization of the Ohio College of Dental Surgery; while among the chief participants in the establishment of the Pennsylvania College of Dental Surgery were Elisha Townsend, Ely Parry, Robert Arthur, J. F. B. Flagg and T. L. Buckingham, all of whom were members of its original faculty and men whose professional eminence and admirable personal qualities gave distinction to the school.

May each of these our elder colleges round out a century of service, and "honor, love, obedience, troops of friends" accompany their ripening years."—Dr. Litch, in July *Dental Brief*.

**INVESTIGATE THE CRITICISMS.** Within the last few months there have been placed before the dental profession two or three new dental journals, which apparently mean to discuss certain phases of the theory and practice of dentistry, as well as some of the political conditions which seem to be rankling in the minds of some of our worthy brethren.

*The Dentist's Magazine*, edited and published by our Cleveland friends, is a worthy contribution to the literature and the better education of the dental profession. *The National Dental Critic* has put forth two issues which indicate that either there is something radically wrong in Washington, the capitol of this great country, or that there is something wrong in the politics of our National Dental Association.

Dr. Emory A. Bryant, editor and publisher, has put in print some material which, if true, places one of the blackest marks upon the quality and sincerity of an organization which should stand for the highest and best in dentistry throughout the entire world. If the gentlemen accused are guiltless of these accusations, means should be found at once to keep such publications from getting into the hands of the general profession. If Dr. Bryant's statements are proven, the dental profession of this country should at once withdraw all support from the National Dental Association. If the *National Dental Critic* is correct in its statement, the story that was told last year that the presidency of the National Dental Association was handed out for certain work in the International Dental Congress in St. Louis may be true. Unfortunately, the organization known as the National Dental Association has been run very largely for the personal aggrandizement of a certain few.

It is interesting to note the career of a number of individuals who have constantly kept their hands upon the throttle of the National Dental Association, and not only in this association, but also in their State and local organizations. The science of politics, if honorable and for the betterment of all parties concerned, is the wisest and most efficient science that man with brains can take up and follow, but when conducted in the manner that the *National Dental Critic* seems to suggest in the National Dental Association, it is a disgrace to the profession of dentistry throughout the civilized word. If these men who are accused by Dr. Bryant don't come out and make clear the falsification of the accusation against them, I, for one, am in favor of advocating absolute desertion of the National Dental Association by the profession, because no man with a particle of respect for his profession should ever countenance or allow such literature to be circulated to the world.

We oftimes hear men complain that there is too much politics in our professional societies, and it is up to the dental profession to at once adopt means and measures to completely demolish either the *National Dental Critic* or the National Dental Association; for it is a disgrace to the dental profession to have such literature thrown broadcast. If these things are not true, they should at once be branded as false.

The dental profession has gained respect from the general public, and the good, substantial part of the dental profession is becoming more and more to look upon these so-called big guys as simply soap bubbles. Dear old Dr. Taft, and men of his stamp, seem to have left the reins of the profession in the hands of many who are incompetent, from a sincere standpoint, and incapable of holding up and making American dentistry continue in its upward course, as it has in years gone by.—G. W. Cook, in June *American.*

